# Regulatory polymorphisms in the bovine *Ankyrin 1 *gene promoter are associated with tenderness and intramuscular fat content

**DOI:** 10.1186/1471-2156-11-111

**Published:** 2010-12-15

**Authors:** Ozlem Aslan, Torres Sweeney, Anne Maria Mullen, Ruth M Hamill

**Affiliations:** 1 Teagasc, Food Research Centre, Ashtown, Dublin 15, Ireland; 2 University College Dublin (UCD), School of Agriculture, Food Science & Veterinary Medicine, Dublin 4, Ireland

## Abstract

**Background:**

Recent QTL and gene expression studies have highlighted ankyrins as positional and functional candidate genes for meat quality. Our objective was to characterise the promoter region of the bovine *ankyrin 1 *gene and to test polymorphisms for association with sensory and technological meat quality measures.

**Results:**

Seven novel promoter SNPs were identified in a 1.11 kb region of the *ankyrin 1 *promoter in Angus, Charolais and Limousin bulls (n = 15 per breed) as well as 141 crossbred beef animals for which meat quality data was available. Eighteen haplotypes were inferred with significant breed variation in haplotype frequencies. The five most frequent SNPs and the four most frequent haplotypes were subsequently tested for association with sensory and technological measures of meat quality in the crossbred population. SNP1, SNP3 and SNP4 (which were subsequently designated regulatory SNPs) and SNP5 were associated with traits that contribute to sensorial and technological measurements of tenderness and texture; Haplotype 1 and haplotype 4 were oppositely correlated with traits contributing to tenderness (*P *< 0.05). While no single SNP was associated with intramuscular fat (IMF), a clear association with increased IMF and juiciness was observed for haplotype 2.

**Conclusion:**

The conclusion from this study is that alleles defining haplotypes 2 and 4 could usefully contribute to marker SNP panels used to select individuals with improved IMF/juiciness or tenderness in a genome-assisted selection framework.

## Background

Ankyrins are structural proteins forming a fundamental component of the cytoskeleton. They are a family of proteins that link the integral membrane proteins to the underlying spectrin-actin cytoskeleton, which in muscle forms a highly complex network of inter-myofibril connections as well as connections between myofibrils and the sarcolemma [1,2] and the sarcoplasmic reticulum [2-5]. Ankyrin contains a regulatory domain which is sensitive to proteolysis [6]. Ankyrins are a known target of the calpains [7-9] which are a family of endogenous cysteine proteases that have been shown to be fundamental to the post-mortem proteolysis of myofibrillar proteins and cytoskeletal proteins and consequent meat tenderisation [10-14]. Degradation of key myofibrillar proteins and the cytoskeleton results in the linkages between myofibrils being broken, thus meat becomes tender [15]. The proteolysis of ankyrin is anticipated to cause a serious effect on cellular integrity [16] with potential consequences for sensory and technological characteristics of meat.

Recent research has highlighted *ANK1 *as a positional and functional candidate gene for meat quality in food-producing animals. The bovine *ankyrin 1 *gene has been mapped close to a QTL for fat associated traits, including marbling in meat [17-19] on bovine chromosome 27. In parallel, expression of the porcine *ankyrin 1 *gene has been associated with a number of meat quality traits, including tenderness, intramuscular fat level (IMF) and water-binding capacity [20].

Genetic polymorphism in the promoter region is one of the mechanisms known to influence variability in the expression of a gene, with associated consequences for the phenotypic diversity of a population [21-25]. Here, the objective of this study was to characterise single nucleotide polymorphisms in a 1.11 kb region of the bovine *ANK1 *promoter region and to test for association with technological and sensory parameters of meat quality traits.

## Results

### Characterisation of a novel muscle transcript of the bovine *ankyrin 1 *gene

**Figure 1:**
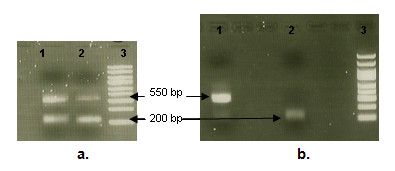
**Identification of alternatively spliced *ANK1 *cDNA transcripts in *M. longissimus thoracis et lumborum***. a. Two transcripts differing in size by approximately 341 bp were identified. b. Two purified transcript variants of *ANK1 *gene. Amplified segments of cDNA from two individuals may be seen in lanes 1 and 2 (amplified using an internal primer from known exon 2); DNA ladder (GeneRuler™ 100-5000 bp, Fermentas) is in lane 3.

**Figure 2:**
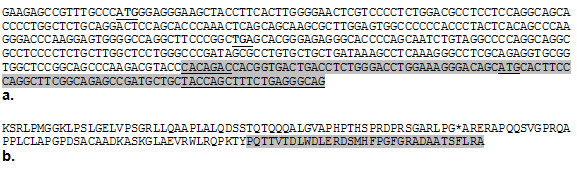
**Identification of the complete 5' end region of a novel muscle transcript of the *ankyrin 1 *gene following 5' RACE-PCR, cloning and sequencing**. a. Newly identified transcription start site (TSS), start codon (ATG) and stop codon (TGA) are in bold and underlined. Alternative, previously published TSS and ATG (from GenBank: AF222766.1) are underlined. The previously known exon1 is shaded and the internal primer is also underlined. b. Protein sequence of the complete 5'end region of a novel muscle transcript. Recognised protein sequence is shaded.

After 5' RACE-PCR, two *ANK1 *transcripts differing in size by approximately 350 bp were observed (Figure 1). The smaller fragment was consistent with previously published sequence [*Bos taurus ankyrin 1 *mRNA, partial CDs; GenBank: AF222766.1]. The larger transcript was novel and hence was cloned and sequenced (Figure 2). The novel mRNA sequence was submitted to the EMBL database with accession number [FR717674].

**Figure 3:**
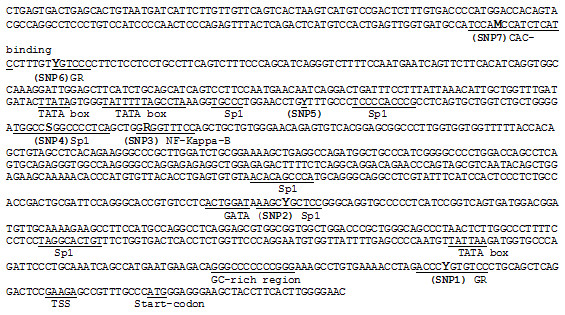
**Nucleotide sequence of the regulatory region 1.11 kb upstream of the novel bovine *ANK1 *transcription start site**. Some of the putative transcription factor binding sites, the TSS, and the predicted start codon are indicated.

341 bp of the larger transcript was not annotated in the NCBI database and hence was considered a novel mRNA sequence. The novel transcription start site (TSS) was upstream of the previously predicted TSS for this gene (39,124,124) reported in [AF222766.1] (btau_4.0). The genomic location of the novel mRNA sequence was immediately adjacent to the known genomic DNA sequence of exon 1. An ATG codon was located 17-19 bp downstream of the novel TSS. The sequence around this new ATG codon matches important elements of the Kozak consensus sequence, including a G at +4, and CC at -2 to -1. In addition, three putative TATA boxes were located 110 bp, 764 bp and 774 bp upstream of the TSS: TTATTAA, TATTTTTA, and TTATA respectively (Figure 3). The predicted initiator methionine was in frame with the previously identified start codon in exon 1. A stop codon was present at position 191-193 bp.

### Promoter SNP discovery

Resequencing of 1.11 kb upstream of the novel bovine *ANK1 *novel transcript TSS revealed seven novel promoter SNPs located as follows, relative to the transcription start site: SNP1 (C:T) at position -24, SNP2 (C:T) at position -307, SNP3 (G:A) at position -672, SNP4 (C:G) at position -687, SNP5 (C:T) at position -733 and SNP6 (C:T) at position -944, and SNP7 (A:C) at position -961 (Table 1 and 2). SNPs 1-5 were observed in all the populations examined and genotype frequencies were in agreement with Hardy-Weinberg equilibrium (Table 2).

**Table 1:** Positions and minor allele frequencies of the rSNPs observed in crossbred cattle (n = 141) and Angus (n = 15), Charolais (n = 15) and Limousin (n = 15) breeds

SNP	SNP position relative to TSS	dbSNP submitter snp (ss) no	Alternate alleles (1/2)	Minor Allele Frequency			
				***Crossbred animals***	***ANG***	***CHAR***	***LIM***
SNP1	-24	275515731	C/T	0.245 (T)	0.100 (T)	0.233 (T)	0.300 (T)
SNP2	-307	275515732	C/T	0.436 (C)	0.400 (T)	0.433 (C)	0.500 (T)
SNP3	-672	275515733	A/G	0.227 (A)	0.100 (A)	0.233 (A)	0.333 (A)
SNP4	-687	275515734	C/G	0.142 (G)	0.100 (G)	0.167 (G)	0.033 (G)
SNP5	-733	275515735	C/T	0.241 (T)	0.100 (T)	0.233 (T)	0.300 (T)
SNP6	-944	275515736	C/T	0.082 (T)	0.0 (T)	0.067 (T)	0.0 (T)
SNP7	-961	275515737	A/C	0.014 (C)	0.0 (A)	0.167 (A)	0.267 (A)

ANG: Aberdeen Angus, CHAR: Charolais, LIM: Limousin

**Table 2:** Observed and expected heterozygosities of the rSNPs in crossbred animals and Angus, Charolais and Limousin breeds

SNP	SNP position relative to TSS	Alternate alleles (1/2)	Crossbred animals (n = 141)		ANG (n = 15)		CHAR (n = 15)		LIM (n = 15)	
			***H_O_***	***H_E_***	***H_O_***	***H_E_***	***H_O_***	***H_E_***	***H_O_***	***H_E_***
SNP1	-24	C/T	0.39	0.37	0.2	0.18	0.333	0.358	0.47	0.42
SNP2	-307	C/T	0.489	0.492	0.533	0.48	0.333	0.491	0.6	0.5
SNP3	-672	A/G	0.355	0.351	0.2	0.18	0.333	0.358	0.4	0.444
SNP4	-687	C/G	0.213	0.243	0.2	0.18	0.2	0.278	0.07	0.064
SNP5	-733	C/T	0.397	0.366	0.2	0.18	0.333	0.358	0.47	0.42
SNP6	-944	C/T	0.163	0.15	0	0	0.133	0.124	0	0
SNP7	-961	A/C	0.028	0.028	0	0	0.333	0.278	0.53	0.391

ANG: Aberdeen Angus, CHAR: Charolais, LIM: Limousin *H_O_*: Observed heterozygosity, *H_E_*: Expected heterozygosity

Allele and haplotype frequencies were determined in two European breeds (Charolais and Limousin) and one British breed (Angus), which are all represented in the Irish herd. SNP6 was monomorphic in Aberdeen Angus and Limousin breeds, while SNP7 was monomorphic in the Aberdeen Angus breed (Table 1, 2). However, only this latter SNP (SNP7) displayed significant divergence in allele frequency between Limousin and Aberdeen Angus (*P *< 0.001) and between Charolais and Aberdeen Angus (*P *= 0.042). SNP1-5 had minor allele frequencies greater than 10% in all populations examined, with the exception of SNP4, which had a minor allele frequency of 3.3% in the Limousin breed (Table 1).

### Transcriptional Effects of regulatory SNPs in the bovine *ANK1 *promoter region

If a transcription factor binding site (TFBS) of a gene is polymorphic in a population this can lead to variation in the level or timing of gene expression [26-28]. In this study, seven novel promoter SNPs were analysed *in silico *to determine if they created or abolished putative transcription factor binding motifs and hence could be described as regulatory SNPs (rSNPs). rSNPs 1, 3, 4 and 7 were located within putative transcription factor binding motifs and either created or abolished putative TFBS as illustrated in Table 3. While three different TFBS predictor tools were used in this study, only five motif changes were supported by more than one prediction tool:

**Table 3:** Selected transcription factor binding motifs in the ANK1 promoter predicted by TESS, TRANSFAC and MatInspector prediction tools

*SNPs	Putative Transcription Factors	Prediction tool	Created with	Recognition Sequence	Start	End
rSNP1 (-24), C/T	GAL4	TESS	C	CCCC	-27	-24
	
	E-box binding factors	MatInspector	C	AGACCCCGTGTCC	-36	-27
	
	USF	TRANSFAC MatInspector	C	CCCCGTGTCC	-27	-18
	
	GR	MatInspector TRANSFAC TESS	T	CAGGGACACAGGGTCTAGG* ACCCTGTGTCC TGTGTC	-15 -28 -24	-33 -18 -19

pSNP2 (-307), C/T	T-Ag	TESS	C	GCCGC	-309	-305
	
	Sp1	TRANSFAC	T	AAGCTGCTCC	-311	-302

rSNP3 (-672), A/G	Sp1	TRANSFAC TESS	A	GCCAGGCCCCTCAGC AGGCCCC	-675 -672	-661 -666
	
	NF-1	TESS	A	TGGA	-675	-672
	
	NF-Kappa-B	TRANSFAC	G	GGGGGTTTCC	-674	-665
	
	GAL4	TESS	G	GGGG	-674	-671
	
	MIG1	TESS	G	CTGGGG	-676	-671

rSNP4 (-687), C/G	Sp1	TRANSFAC TESS	C	ATGGCCCGGCCCCT GCCCGGCCCC	-666	-669
					
					-690	-681
	
	Oct-01	TRANSFAC	C	GCCCGGCCCC	-690	-681
	
	NF-1	TRANSFAC	C	TGGCCGGGCC	-691	-682
	
	Sp1	TRANSFAC	G	GGCCGGGCCCCTCA	-691	-678
	
	YY1	TRANSFAC TESS	G	GATGGCCGG	-694	-686
	
	GCF	TESS	G	SCGSSSC	-689	-683
	
	Krueppel like	MatInspector	G	CTGAGGGGCCGGGCCAT*	-677	-693
	
	Nuclear factor Kappa B/C-Rel	MatInspector	G	TGGGGATGGCCCG	-698	-686
	
	GC-box factors SP1/GC	MatInspector	G	GAGGGGCCGGGCCAT*	-679	-693

pSNP5 (-733), C/T	Oct-01	TRANSFAC	C	TGCTTTGCCC	-737	-728
	
	Lvc	TESS	C	CCTGC	-737	-733
	
	GCF	TESS	C	GSSSCGS	-737	-731
	
	Sn	TESS	T	ACCTGTT	-738	-732
	
	Grainyhead-like factors	MatInspector	T	GGAACCTGTTTTG	-741	-729

pSNP6 (944), C/T	Oct-01	TRANSFAC	C	CTGCTTTGCC	-947	-938
	
	Glucocorticoid receptor	TESS	T	TGTTGT TGTCCC	-947 -944	-942 -939
	
	Krueppel like factors	MatInspector	T	CAACAAAGGATGAGATG*	-943	-959

rSNP7 (961), A/C	CAC-binding	TESS MatInspector	C	CCACC TCCACCCATCTCATC	-964 -965	-960 -651

*Only the SNPs that create putative transcription factor binding sites were listed. *Minus strand. SNP positions in the recognition sequence are underlined.

#### rSNP1

Glucocorticoid receptor- The presence of the minor T allele at rSNP1 led to the creation of a putative glucocorticoid receptor transcription factor binding site, which was not present with the alternate C allele.

#### rSNP4-Sp1

The presence of the minor G allele at rSNP4 abolishes a putative Sp1 transcription factor binding site, that was present with the alternate.

#### rSNP4-delta factor (YY1)

The presence of the minor G allele created a delta factor transcription factor binding site, that was not present with the alternate.

#### rSNP7-CAC-binding

The presence of the minor C allele at rSNP7 creates a putative CAC-binding transcription factor binding site, which was not present with the alternate A.

### Haplotype construction

**Figure 4:**
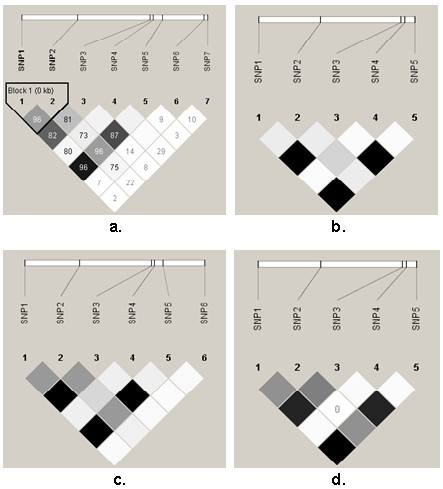
**Pairwise linkage disequilibrium plots indicating *r^2 ^*values for 7 SNP in the regulatory region of the examined populations**. a. Crossbred samples (n = 141), b. Aberdeen Angus, c. Charolais, d. Limousin (n = 15 for each breed). Dark grey and black squares indicate statistically significant (LOD > 2) allelic association between a pair of SNPs; darker greys indicate higher values of ***r^2^***, up to a maximum of 1 (black). White and light grey squares indicate no statistically significant evidence of LD.

Linkage disequilibria plots are presented in Figure 4a-d. Pair-wise *r^2 ^*values were close to 1 for rSNP1, 3 and 5, indicating they were in high linkage disequilibrium with each other in the four populations. Haplotypes are defined as a combination of SNP alleles closely linked on the same chromosome. Haplotype-based methods can provide higher power to asses the complex relationship between genetic variation and phenotypes, compared with methods based on single SNP analysis [29,30].

**Figure 5:**
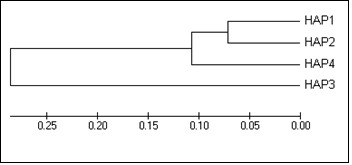
**Neighbour joining tree of four major *ANK1 *promoter haplotypes in crossbred cattle**. HAP1 (CTGGCCA) and HAP2 (CTGCCCA) were the most closely related haplotypes, followed by HAP4 (CCGCCCA). HAP3 (TCACTCA) was the most distant.

Eighteen potential haplotypes were inferred in the study. Four inferred haplotypes had allele frequencies greater than 10% (Table 4) in the crossbred animals group. The top three haplotypes were HAP2 (CTGCCCA), HAP3 (TCACTCA) and HAP4 (CCGCCCA) with frequencies of 0.344, 0.185 and 0.177 respectively. These were also the top three haplotypes in both Charolais and Limousin breeds. In the Aberdeen Angus breed, the main haplotypes were HAP1 (CTGGCCA), HAP2 (CTGCCCA) and HAP4 (CCGCCCA) which had frequencies of 0.10, 0.266 and 0.534 respectively. Phylogenetic reconstruction indicated that HAP1 and HAP2 were the most closely related haplotypes, followed by HAP4. HAP3 was most distant (Figure 5).

**Table 4:** Haplotype frequencies for crossbred animals and Angus, Charolais and Limousin breeds

**HAP ID	Haplotype	Crossbred animals (n = 141)	ANG (n = 15)	CHAR (n = 15)	LIM (n = 15)
1	CTGGCCA	0.123	0.100	0.167	-
2	CTGCCCA	0.344	0.266	0.333	0.500
3	TCACTCA	0.185	0.066	0.233	0.300
4	CCGCCCA	0.177	0.534	0.200	0.133

**Only haplotypes with a frequency greater than 10% of population are presented

ANG: Aberdeen Angus, CHAR: Charolais, LIM: Limousin

### Association analysis

#### Single SNP association

Associations between the rSNPs and meat quality traits (technological and sensory) are reported in Table 5. Least-squares means for meat quality traits for each genotype are also presented in Table 5. Overall, four of the five single SNPs tested showed association with at least one meat quality trait. These comprised of 17 significant associations (*P *< 0.05) and 6 tendencies towards association (*P *< 0.1) in total. Three SNPs which were in strong LD with each other (rSNP1, rSNP3 and rSNP5) were associated with sensory tenderness in LTL and/or SM muscle. rSNP1 and rSNP5 were also associated with an objective measure of tenderness (Warner-Bratzler shear force) in both LTL and SM muscles, while rSNP3 was associated with shear force in LTL. rSNP1 and rSNP5 were also associated with sensory texture in both LTL and SM muscles, whereas rSNP3 and rSNP4 were associated with texture in LTL and SM muscle, respectively.

**Table 5:** Significant associations (P < 0.05) and tendencies towards association (P < 0.1) between rSNP genotypes and meat quality traits and least squares means for each genotype

SNPs	Traits	*P*-value	Least squares mean per genotype		
			*1/1*	*1/2*	*2/2*
			n = 79	n = 55	n = 7
			
rSNP1	Tenderness-SM	**0.0002**	5.38 ± 0.14^a^	4.77 ± 0.14^b^	4.93 ± 0.36^ab^
	Shear force-SM	**0.0258**	52.05 ± 1.75^a^	56.94 ± 1.75^b^	55.13 ± 4.41^ab^
	Shear force-LTL	**0.0336**	44.38 ± 2.92^a^	52.00 ± 2.92^b^	50.20 ± 6.78^ab^
	Firmness-SM	0.0628	5.52 ± 0.09^a^	5.74 ± 0.09^b^	5.59 ± 0.22^ab^
	Texture-SM	**0.0015**	3.33 ± 0.09^a^	3.67 ± 0.09^b^	3.80 ± 0.23^ab^
	Texture-LTL	**0.0152**	3.10 ± 0.11^a^	3.42 ± 0.11^b^	3.01 ± 0.26^ab^
	Juiciness-SM	0.0713	4.84 ± 0.14	4.50 ± 0.14	4.69 ± 0.36
	Juiciness-LTL	0.0970	5.33 ± 0.15	5.09 ± 0.15	4.72 ± 0.35

			n = 27	n = 69	n = 45
			
pSNP2	Texture-SM	0.0996	3.60 ± 0.13	3.57 ± 0.09	3.34 ± 0.11
	Texture-LTL	0.0827	3.12 ± 0.15	3.38 ± 0.11	3.14 ± 0.13

			n = 7	n = 50	n = 84
			
rSNP3	Tenderness-SM	**0.0460**	5.19 ± 0.37^ab^	4.87 ± 0.15^a^	5.27 ± 0.15^b^
	Tenderness-LTL	**0.0493**	5.60 ± 0.37^ab^	5.20 ± 0.16^a^	5.63 ± 0.16^b^
	Shear force-LTL	**0.0051**	50.67 ± 6.63^ab^	52.88 ± 2.89^a^	43.06 ± 2.94^b^
	Texture-LTL	**0.0021**	2.88 ± 0.26^a^	3.46 ± 0.11^b^	3.06 ± 0.26^ab^
	Flavour-SM	0.0496	3.94 ± 0.13	3.66 ± 0.05	3.78 ± 0.05

			n = 136	n = 30	n = 5
			
rSNP4	Texture-SM	**0.0006**	3.46 ± 0.08^a^	3.74 ± 0.11^b^	2.81 ± 0.24^c^
	Firmness-SM	0.0753	5.60 ± 0.08	5.76 ± 0.11	5.25 ± 0.23
	Firmness-LTL	**0.0479**	5.18 ± 0.09	5.38 ± 0.13	5.78 ± 0.27

			n = 79	n = 56	n = 6
			
pSNP5	Tenderness-SM	**0.0037**	5.34 ± 0.14^a^	4.83 ± 0.14^b^	5.23 ± 0.40^ab^
	Shear force-SM	**0.0084**	51.54 ± 1.76^a^	57.13 ± 1.71^b^	54.78 ± 4.84^ab^
	Shear force-LTL	**0.0038**	43.07 ± 2.92^a^	52.49 ± 2.82^b^	53.77 ± 7.25^ab^
	Texture-SM	**0.0279**	3.37 ± 0.09^a^	3.63 ± 0.09^b^	3.61 ± 0.27^ab^
	Texture-LTL	**0.0044**	3.09 ± 0.11^a^	3.43 ± 0.11^b^	2.78 ± 0.28^ab^

Significant associations are in bold and associations remaining significant after Bonferroni correction are underlined (Bonferroni-adjusted *P*-value = 0.0014). Least squares means with different superscripts had significant Tukey-Kramer contrasts. Genotype categories (1/1, 1/2, 2/2) relate to the alternate alleles designated in Table 1-2. n: number of samples with each rSNP genotypes.

LTL: M. *longissimus thoracis et lumborum *muscle; SM: M. *semimembranosus *muscle

#### Haplotype association

A number of significant associations were found between individual *ANK1 *promoter haplotypes and aspects of meat quality. Least squares means, associated *P*-values and Tukey-Kramer contrasts are presented in Table 6. Each of the four most common haplotypes was associated with at least one aspect of sensory or technological quality:

**Table 6:** Significant associations (P-value < 0.05) and tendencies towards association (P-value < 0.1) for the most abundant ANK1 gene promoter haplotypes with meat quality traits and least squares means for haplotype copy number

HAP ID	Trait	*P*-value	Estimated trait mean per haplotype copy		
			0	1	2
HAP1			N = 109	N = 27	N = 5
			
	Texture-SM	**0.0012**	3.48 ± 0.08^a^	3.74 ± 0.12^a^	2.82 ± 0.24^b^
	Texture-LTL	0.0974	3.18 ± 0.10	3.40 ± 0.15	3.74 ± 0.30
	Firmness-SM	0.0917	5.61 ± 0.08	5.76 ± 0.11	5.25 ± 0.23
	Firmness-LTL	**0.0176**	5.18 ± 0.09	5.45 ± 0.13	5.79 ± 0.26

HAP2			N = 61	N = 67	N = 13
			
	Texture-SM	**0.0125**	3.66 ± 0.09^a^	3.39 ± 0.09^b^	3.36 ± 0.19^ab^
	Texture-LTL	0.0861	3.33 ± 0.11	3.21 ± 0.11	2.82 ± 0.23
	Juiciness-SM	0.0918	4.51 ± 0.14	4.83 ± 0.14	4.78 ± 0.29
	IMF-SM	**0.0275**	1.06 ± 0.16^a^	1.10 ± 0.16^a ^	1.95 ± 0.33^b^
	IMF-LTL	**0.0276**	2.06 ± 0.24^a ^	2.41 ± 0.24^ab^	3.30 ± 0.48^b^

HAP3			N = 92	N = 43	N = 6
			
	Tenderness-SM	**0.0317**	5.26 ± 0.14^a^	4.82 ± 0.16^b^	5.20 ± 0.41^ab^
	Shear force-LTL	0.0727	45.19 ± 2.90	51.83 ± 3.11	54.42 ± 7.44
	Texture-LTL	**0.0070**	3.12 ± 0.11^a^	3.46 ± 0.12^b^	2.80 ± 0.29^ab^

HAP4			N = 100	N = 35	N = 6
			
	Tenderness-LTL	**0.0195**	5.30 ± 0.14^a^	5.82 ± 0.20^b^	5.65 ± 0.42^ab^
	Shear force-LTL	**0.0188**	50.12 ± 2.61^a^	41.99 ± 3.57^b^	36.92 ± 7.68^ab^
	Texture-LTL	**0.0146**	3.34 ± 0.10^a ^	2.97 ± 0.14^b^	2.99 ± 0.30^ab^
	Firmness-LTL	**0.0143**	5.34 ± 0.09^a ^	5.00 ± 0.12^b^	5.02 ± 0.27^ab^

Significant associations are in bold and associations remaining significant after Bonferroni correction are underlined (Bonferroni-adjusted *P*-value = 0.0014). Least squares means with different superscripts had significant Tukey-Kramer contrasts. N (Number of samples with each haplotype copies)

LTL: M. *longissimus thoracis et lumborum *muscle; SM: M. *semimembranosus *muscle

##### HAP1

Homozygous HAP1 individuals had reduced texture scores in SM (*P *= 0.0012) and increased firmness scores in LTL muscle (*P *= 0.0176), compared to individuals with no copy of this haplotype.

##### HAP2

The most common haplotype, HAP2, was associated with IMF content in both LTL and SM muscle (*P *= 0.0276 and *P *= 0.0275, respectively). Homozygous HAP2 individuals had almost twice as much IMF content (1.95%) in SM, compared with those that had no copies of the haplotype (1.06%). In LTL muscle, there was also an increase in IMF % with haplotype 2. IMF levels were higher in LTL muscle, compared with SM but the trend was the same - Individuals with two copies had 3.24% fat compared with 1.99% for those with zero copies. For both SM and LTL, individuals with one copy of the haplotype had intermediate levels of fat. This association was supported by a tendency towards increased juiciness in SM muscle (*P *= 0.077) of HAP2, as fat influences juiciness [31,32].

##### HAP3

This haplotype was associated with texture in LTL (*P *= 0.0070) and tenderness score in SM muscle (*P *= 0.0317).

##### HAP4

A significant observation was that homozygous or heterozygous individuals for HAP4 had increased tenderness scores, decreased firmness and lower shear force values in LTL muscle compared to non-carriers. In addition, non-carrier animals were 10% firmer (5.34 **± **0.09) than for either homozygous or heterozygous carriers (5.02 **± **0.27 and 5.00 **± **0.12, respectively). Individuals with one copy of HAP4 had lower texture score compared to those without this haplotype.

##### HAP5

This haplotype was associated with texture in SM muscle (*P *= 0.0445). Heterozygous individuals had increased texture score compared to those with no copy of this haplotype.

## Discussion

Here, we have identified a new transcript variant of the bovine *ankyrin 1 *gene in muscle. Seven novel SNPs were identified in the promoter region and several associations between the promoter SNPs and haplotypes and meat quality traits were observed. After correction for multiple testing, associations between rSNPs 1 and 4, and haplotype 2 and meat quality were significant.

We have shown that a novel variant of the *ankyrin 1 *gene is present in muscle. This variant is longer than previously reported variants (NCBI) and increases the overall gene length by 341 bp in the 5' direction. The identification of alternative transcripts of bovine *ANK1 *is not surprising. Indeed, mammalian genomes express three ankyrin proteins, which are encoded by three separate genes (*ANK1-3*) [33-35]. In bovine, *ANK2 *is located on chromosome 6 (13,335,381 - 13,706,921; btau_4.0) and *ANK3 *is located on chromosome 28 (14,417,416 - 14,535,840; btau_4.0). Unlike *ANK1*, neither map close to QTL for meat quality traits. However, *ANK2 *maps close to several QTL for carcass yield traits including *longissimus *muscle area [18] and back fat EBV [36,37]. The functional and molecular diversity of the ankyrin family is a result of differential expression of these three independent genes and their widely alternatively spliced variants, some missing large segments that include whole functional domains [38-42]. It has been reported that *ankyrin 1 *has the most limited pattern of expression restricted to erythroid, muscle and neural tissue in human, but despite these limitations, tissue-specific isoforms with multiple transcripts are generated by alternative splicing under the control of several tissue-specific promoters [33,39].

Single nucleotide polymorphisms (SNPs) within regulatory regions can regulate the level and/or timing of gene transcription [26-28]. Evolutionary mutations arising in a promoter can have variable effects depending on the rSNP location relative to TFBS, start codons, transcription start sites etc, and the intracellular molecular environment. In some cases, a SNP may eliminate the natural binding site of a tissue-specific transcription factor or create a new binding site [28]. Therefore, functional rSNPs in TFBS may lead to differences in gene expression [28] with significant potential to increase phenotypic diversity. In this study, several *ANK1 *rSNPs were associated with meat quality traits. After correction for multiple testing, two of the SNP associations remained significant. These were the association between rSNP1 and tenderness score and the association between rSNP4 and texture values. While it is possible that these are marker SNPs rather than causative, of interest was that the rSNPs were found within well supported putative transcription factor binding motifs. For instance, presence of the minor T allele of rSNP1 created a glucocorticoid receptor (GR) motif and presence of the C allele of rSNP4 created a Sp1 motif. Glucocorticoids are steroid hormones which regulate a variety of biological processes and can influence many functions of a cell [43]. The GR functions as a hormone-induced transcription factor which has been shown to regulate (either stimulate or repress) gene expression [43-45]. The presence of allele C of rSNP4 led to the creation of a Sp1 motif. Sp1 has been shown to bind to a consensus sequence of the leptin gene promoter and mutations in this region abolished the binding and reduce promoter activity in rat [46]. Adamowicz and co-workers [47] also reported that a mutation in the Sp1 binding site of the bovine leptin gene affects its expression level. The presence of a G allele of this SNP (rSNP4) created a binding site for the zinc finger nuclear protein, YY1 (Yin Yang 1), also known as NF-E1, UCRBP or CF1, which is known to have fundamental roles in repressing and activating a diverse group of promoters; it not only initiates transcription but also activates or represses it [48,49]. YY1 is also shown to repress muscle specific expression of sarcomeric alpha-actin and c-myc genes [49]. Walowitz and co-workers also reported that calpains and the 26 S proteasome are involved in YY1 stability in rat muscle indicating that myogenic transcription may be inactivated by developmentally regulated proteolysis to promote muscle development. While it was not the aim of the present study, further functional studies would be valuable to assess the role of these TFBS, in modulating the expression of muscular transcripts of the bovine *ankyrin 1 *gene.

Few studies have examined variation in *ANK1 *in relation to muscle biology. However, Wimmers et al. [20] examined one SNP in the 3' UTR region of the porcine homolog. They have reported that this SNP in the 3' UTR of porcine *ANK1 *showed associations with shear force and IMF content, as well as several traits related to water-binding capacity in muscle [20], in several pig breeds.

In the present study, several of these rSNP were linked, therefore association analysis with individual haplotypes was also carried out. HAP4 was significantly associated with measures of tenderness. Sensory tenderness and firmness scores were improved in animals with HAP4 genotypes (approximately 5-10%), as was shear force (10-20%). These effect sizes suggest that the influence on tenderness is quantitative rather than that of a major gene [50]. However, this effect may be important to perceived tenderness. Recent research has shown that beef with shear force values higher than 52.68 N can be reliably classified as 'tough' by sensory panellists and beef with shear force values lower than 42.87 N can be reliably classified as 'tender' [51]. The observed genotypes and haplotypes at these promoter SNPs can thus potentially discern 'tender' from 'intermediate' to tough' beef. The findings reported here may be significant as there are to date, few markers for tenderness that have been widely confirmed and are in commercial use e.g. SNP in *calpain *and *calpastatin *gene regions [52-54] and expression level of *DNAJA1 *[55,56].

This haplotype (HAP4) was, however, associated with lower scores for texture. This means that selection for improved tenderness may result in a disimprovement in texture. However, it should be noted that sensory texture profile is not as well correlated with sensory tenderness as is Warner Bratzler shear force [57]. Low values of texture can be scored for meat that is too soft and mushy or too firm and unyielding. In contrast, low scores of tenderness and firmness are clearer in their meaning to sensory panellists (tougher and less firm respectively). Additionally, we also observed that homozygous HAP1 individuals also had reduced texture scores in SM muscle compared to individuals with no copy of this haplotype. This association remained significant even after a stringent Bonferroni correction (*P *= 0.0012), suggesting a possible role in influencing bovine muscle texture. Homozygous individuals with this haplotype also had increased firmness scores in LTL muscle.

Post-mortem tenderisation is influenced by enzymatic degradation of myofibrillar and associated cytoskeletal proteins. These proteins function to maintain myofibrillar integrity and it is known that the calpain system, in particular calpain 1, is responsible for proteolysis of these proteins [10,12,13,15]. The calpain family of Ca^2+^-dependent proteases are thus key proteins in the regulation of tenderness [58-60]. Proteins which are reported to be proteolysed by calpains can be classified as kinases/phosphatases, membrane associated proteins, some transcription factors and cytoskeletal proteins which include desmin, gelsolin, myosin, nebulin, spectrins, titin, tropomyosin -for review see [61]. Ankyrin is one of the cytoskeletal proteins proteolysed by members of the calpain family [7,16,61,62]. Since ankyrin has functions in maintaining the structural integrity of, and linkages among myofibrils, the degradation of ankyrin could cause further softening of myofibrils and thus, tenderization. The relationship between tenderness (sensory and shear force) and HAP4 or rSNP1, rSNP3 of the bovine *ANK1 *promoter, may indicate that this haplotype and/or these rSNP might alter the expression of cytoskeletal protein *ankyrin 1 *in muscle and thus the extent of post-mortem proteolysis by calpains.

An association with IMF content (%) was observed for HAP2 in both muscles. This was supported by sensory juiciness score, which is an indirect sensory measure of intramuscular fat content [31,32], in that HAP2 showed a tendency towards increased juiciness in SM muscle. Intramuscular fat has been shown to contribute 10 - 15% of the variance in palatability of beef [63]. These results suggest that HAP2 may have potential in selection for IMF content. It is curious that HAP2 was associated with IMF but only differs from HAP4 and HAP1 by a single SNP in each case (rSNP2 and rSNP4, respectively). Neither rSNP2 nor rSNP4 are associated with IMF alone. This suggests that the relationship between HAP2 and this trait may be via linkage to nearby functional SNP. In fact, a number of QTL for marbling and fat thickness have been identified on this chromosome in a mapping population of *Bos indicus *× *Bos taurus *(including Hereford, Angus and Charolais) cattle [18,19]. More recently, 3 QTL for marbling and 2 QTL for fat thickness have been identified on chromosome 27 in commercial Angus cattle [19]. While *ANK1 *is not located within any of these QTL, its proximity supports the hypothesis that it is a candidate gene for these traits.

Many improvements in meat quality can be achieved by optimising systems management in animal production and meat processing [64-66]. However, in addition to environmental and processing influences on meat quality, there are certainly biological factors which affect the quality of meat [67-70]. The identification of genomic regions influencing economically important traits, in particular tenderness and intramuscular fat, has been made possible due to polymorphic markers and linkage maps in cattle. However, just a few genes have been located within these QTL and found to be influencing these traits. In this study we have found that polymorphisms (SNPs and haplotypes) of the *ankyrin 1 *gene are in association with these important meat quality traits. This is the first time such associations have been identified, therefore it will be important to confirm these findings in independent populations [71].

## Conclusions

In this report, we have shown a new transcript variant of the bovine *ankyrin 1 *gene in muscle. We have identified seven novel SNPs in the promoter region of bovine *ANK1 *and several of these SNPs and inferred haplotypes were associated with meat quality traits, in particular meat tenderness and intramuscular fat. Of interest was that some of these promoter SNPs were also found within well supported putative transcription factor binding motifs, supporting the assumption that SNPs in transcription factor binding sites may lead to differences in gene expression with significant potential to increase phenotypic diversity. This gene region is a potential target for gene-assisted selection to improve meat quality. However, validation of these associations in independent populations is key before their practical application. Additional future studies could focus on expression analysis to demonstrate the functional role of the identified promoter variation.

## Methods

### Identification of the transcription start site of the bovine *ankyrin 1 *gene

Most of the sequences available in the NCBI and Ensembl databases shared little to no consensus concerning the size of the 5' UTR region of the *ankyrin 1 *gene or of a transcription start site (TSS). Hence, the TSS of the muscle *ANK1 *gene was determined by 5' RACE-PCR.

#### Tissue samples and RNA extraction

*M. longissimus thoracis et lumborum *(striploin) samples were taken from 3 slaughter-weight commercial beef animals within one hour post slaughter in an EU-licensed abattoir. Tissue samples (2 g each) were homogenised and preserved in 10 ml RNAlater^® ^(Ambion, Inc. Austin, TX). Total RNA was extracted from 50 mg of the preserved bovine muscle tissue using TRI Reagent^® ^(Molecular Research Centre, Inc. USA), following the supplier's protocol. The RNA was then dissolved in 40 μl of 0.1% DEPC treated water and then subjected to deoxyribonuclease I (DNase I) treatment (QIAGEN Ltd., West Sussex, UK). Purification of RNA was carried out using the universal phenol-chloroform method. The RNA pellet was finally resolvated in 40 μl of nuclease-free water. Quality and quantity of the total RNA was assessed on a 1.2% agarose gel and on a NanoDrop^® ^ND-1000 Spectrophotometer (Thermo Fisher Scientific Inc. MA, USA), respectively. Samples with an A260/280 ratio 1.7 ≤ 2.0 were selected for further examination. RNA was aliquoted and stored at -80°C prior to cDNA synthesis.

#### 5' end cDNA synthesis and amplification of the 5' end cDNA region

5' end cDNA was synthesized from 2.5 μg of total RNA using a GeneRacer™Kit (Invitrogen Corp., San Diego, CA, USA) according to supplier's protocols. 5' cDNA was synthesized from modified RNA using Superscript™III first-strand synthesis system using oligo(dT)_20 _primer with 1.0 μl of total RNA in a final reaction volume of 20 μl using the Superscript™ III First-strand synthesis system for reverse transcriptase-polymerase chain reaction (RT-PCR) (Invitrogen Corp.). GeneRacer Oligo dT primer with oligo (dT)_24 _with the anchor sequence was used to reverse transcribe the mRNA. A gene specific primer (ANK1E2; reverse: 5'-CTG CCC TCA GAA AGC TGG TA-3', btau_4.0; chr27: 39,084,536 - 39,084,476, minus strand) [GenBank: NC_007328.3] was designed based on the *ANK1 *bovine genomic DNA sequence to amplify different sizes of cDNA fragment from the total RNA extracted. The single-stranded cDNAs were amplified using the 5' adaptor primer (Forward 5'-CGA CTG GAG CAC GAG GAC ACT GA-3') (Invitrogen Corp.). High fidelity Supermix (Invitrogen Corp.) was used for the PCR in a total volume of 25 ml and the conditions were as follows: initial denaturation at 94°C for 2 min followed by 40 cycles of denaturation (94°C 30 s), annealing (56°C for 30 s) and extension (68°C for 30 s) with a final extension at 72°C for 5 min. PCR products were visualized on a 1.2% agarose gel.

Cloning of the extracted 5'end RACE cDNA fragments was achieved by using the TOPO-TA cloning kit (Invitrogen Corp.). cDNAs were ligated into pCR^®^4-TOPO plasmid followed by transformation into TOP10 *Escherichia coli *cells (Invitrogen Corp.). Single colonies were transferred to cell culture plate containing 10 ml LB medium supplemented with 50 mg/ml ampicillin (Sigma-Aldrich Corp., USA) and grown overnight at 37°C, 140 r.p.m. Small aliquots of the overnight cell cultures of recovered colonies were plated at limiting dilution in 15 ml LB containing 100 mg/ml ampicillin. Plasmid DNA was purified using the GenElute^™ ^Plasmid Purification kit (Sigma-Aldrich Corp.) according to supplier's protocol.

30 μl of PCR product was cleaned up using Gen Elute™PCR clean-up kit (Sigma-Aldrich Corp.) following the manufacturer's protocol. The quality and quantity of the purified PCR product was assessed on a 1.2% agarose gel and on a NanoDrop^® ^ND-1000 Spectrophotometer (Thermo Fisher Scientific Inc. MA, USA). Randomly selected 12 plasmid DNAs (200 ng) were sequenced in both reverse and forward directions to confirm constructs by Eurofins, MWG-Biotech (Edelsberg, Germany).

### Animals, DNA preparation and Phenotype

#### Animals and DNA preparation

Blood samples of unrelated purebred Aberdeen Angus, Charolais and Limousin bulls (n = 15 per breed) were taken to determine the base-line level of DNA polymorphisms in purebred beef breeds. *M. longissimus thoracis et lumborum *(LTL) and *M. semimembranosus *(SM) tissue samples of commercial crossbred cattle (n = 141; the major contributing breeds were Charolais, Hereford, Limousin, Friesian, Holstein-Friesian and Simmental) were collected from an EU licensed abattoir and meat quality measured for a number of traits as described previously [72]. A total of seven meat quality traits for each muscle type (SM and LTL) were examined, two relating to technological measure of meat quality i.e. intramuscular fat content and shear force; and five relating to sensory traits i.e. texture, tenderness, flavour, juiciness and firmness. DNA was extracted from muscle and blood using the DNeasy^® ^blood and tissue kit (QIAGEN Ltd.).

#### Phenotype

The meat quality data has been analysed and previously published in detail [70,72,73]. Compositional analysis was carried out on meat frozen at 14 days post-mortem and IMF % levels assessed according to a recognised protocol [74]. The Warner-Bratzler shear force measurement is the most widely accepted instrumental technique to determine the tenderness of meat. Warner-Bratzler shear force values represent the amount of force required to shear a 2.54 cm core of a cooked and tempered meat sample [75], in this study they are reported in Newtons (N). The less force (lower N) required to shear through the meat sample, the more tender the product. Tenderness, texture, flavour and firmness measures were carried out by sensory analysis. The panellists graded tenderness, texture and firmness on a scale of 1-8, as follows: (tenderness 1 = extremely tough, 8 = extremely tender; texture: 1 = very poor, 8 = extremely good; firmness: 1 = extremely mushy, 8 = extremely firm). Panellists graded flavour and juiciness in a scale of 1-6, as follows: flavour: 1 = very poor, 6 = extremely good; juiciness: 1 = very dry, 6 = extremely juicy. Studies have shown that there is a fairly high, but not perfect, inverse correlation (-0.72) between Warner-Bratzler shear force measurements and sensory tenderness ratings [51]. Samples producing shear force values of greater than 52.68 Newtons were considered 'tough' by sensory panellists [51].

### Polymorphism identification and validation

Initially the 1.11 kb promoter region of the *ankyrin 1 *gene was resequenced in all purebred and crossbred animals. Subsequently, three of the SNPs were validated using custom Taqman SNP genotyping assays.

#### DNA sequencing

In order to amplify 1.11 kb of the bovine *ANK1 *promoter two sets of overlapping primers (fragment 1, 702 bp: forward: 5'-CTG AGT GAC TGA GCA CTG TAA TGA-3', reverse: 5'-GGT GTT TTT GCT TCT CCA GC-3' btau_4.0 chr27: 39,125,655 to 39,124,954; fragment 2, 711 bp: forward: 5'-AGC TGC TGT GGG AAC AGA GT-3', reverse: 5'- GTT CCC CAA GTG AAG GTA GC-3' btau_4.0 chr27: 39,125,196 to 39,124,486, minus strand) were designed to detect genetic polymorphisms based on published sequence data [GenBank: NW_001494427.2]. 100 ng of genomic DNA was used to generate PCR products in the presence of 200 pM of both forward and reverse primers. PCR amplifications were performed with 2 μl of the genomic DNA template in a 50 μl final reaction volume. The PCR reaction consisted of 10 μl 5× PCR buffer, 3 μl 25 mM MgCl2, 1 μl dNTPmix (10 mM of each), 2 μl Primer mix (0.2 μM each of the forward and reverse primers), 0.4 μl GoTaq^® ^DNA Polymerase (100 U) (PGEM, Promega Corp. USA). The cycling conditions used were as follows: initial denaturation at 94°C for 2 min followed by 35 cycles of 94°C for 45 sec, 56 and 57°C (respectively for each primer pair) for 45 sec and 72°C for 1 min 20 sec for followed by a final extension of 72°C for 10 min for all fragments. All PCR products were analysed by agarose gel electrophoresis (1.2%) and ethidium bromide staining and visualised using the Epi Chemi II Darkroom (UVP, Upland, CA) equipped with the image analysis program LabWorks. PCR products were purified prior to sequencing using the QIAGEN PCR purification kit (QIAGEN Ltd.). Sequencing of the purified PCR product was carried out by Eurofins, MWG-Biotech. Sequences were aligned and the data analyzed using MEGA^® ^(Molecular Evolutionary Genetic Analysis) v 4.0 software [76] MEGA4.

#### SNP validation

Two closely linked SNPs (*r^2 ^*= 1) (rSNP1 and rSNP5s) and another SNP (rSNP2) were selected and re-genotyped for validation purposes using Custom TaqMan^® ^SNP Genotyping Assays (Applied Biosystems, Foster City, CA, USA), in all commercial crossbred and purebred samples. Primers and probes for the assay were designed using Primer Express and were synthesized by Applied Biosystems. Each 10 μl PCR reaction consisted of 5 μl mastermix (TaqMan^® ^genotyping mastermix, Applied Biosystems), 0.25 μl genotyping assay, 2.75 μl dH2O and 2 μl of DNA (10 ng/μl). The PCR thermal cycling was as follows: initial denaturing at 95°C for 10 min; 40 cycles of 95°C for 15 s and 60°C for 1 min. Thermal cycling was performed using an ABI PRISM^® ^7500.

### Transcriptional effects of regulatory SNPs in the bovine *ANK1 *promoter region

To identify SNPs that affect promoter elements, including putative transcription factor binding sites of interest, 1.11 kb upstream of the *ANK1 *translation start site were screened for the presence of putative selective transcription factor binding sites *in silico *using three prediction tools (TESS; http://www.cbil.upenn.edu/tess), TRANSFAC http://www.biobase-international.com/index.php?id=transfac and MatInspector http://www.genomatix.de/en/index.html. Particular attention was paid to binding sites already proven to be of significance in regulating the muscle specific binding sites of bovine and other common binding sites in mouse and human.

### Statistical methods

#### Allele, Genotype, and Haplotype Frequencies, LD

Estimation of allele frequencies and departures from Hardy-Weinberg equilibrium (HWE) were carried out using the population genetic data analysis software Haploview [77]. Linkage disequilibrium measures were also estimated using Haploview. Following haplotype inference, the ELB algorithm was used to assign the most likely haplotype combination to an individual as implemented in the Arlequin program [78].

#### Association analysis

Evaluation of association between the seven SNPs and values of meat quality traits for each individual SNP in the crossbred population (n = 141) were carried out using the least squares method of the GLM (General Linear Model) procedure in SAS (Version 9.1, SAS Inst., Inc., Cary, NC, 2002-2003). A number of covariates (sex, age, carcass weight, slaughter period, and slaughter factory) were included in the model to control for potentially confounding factors. Five *ANK1 *regulatory SNPs with MAF >0.1 were tested for associations with meat quality traits. Where significant associations were identified, mean trait values for each genotype were contrasted using the Tukey-Kramer procedure in SAS. In order to evaluate the association of individual alleles with meat quality, the four most common haplotypes of the crossbred population (frequencies of more than 10%) were also tested in separate GLM models including the same covariates as the single SNP analyses. Each analysis tested for a difference in least squares means of meat quality traits where 0, 1 or 2 copies of each haplotype were present. Tukey-Kramer analysis was also carried out for significant associations. A Bonferroni-adjusted *P*-value was calculated for individual SNP and haplotype analyses.

## Authors' contributions

OA carried out the laboratory work, population genetic data analysis, participated in data interpretation and prepared the first draft of the manuscript. TS, RMH and AMM designed and coordinated the study. RMH and TS participated in data interpretation and drafting of the manuscript. All authors agreed with the final manuscript.
